# Toward Autonomous Clinics: Human–Robot Collaboration in Clinical Care

**DOI:** 10.1002/mco2.70885

**Published:** 2026-07-25

**Authors:** Xinyuan Wu, Jingrao Zhang, Mengdi Xu, Henry K. Chu, Xiaolan Chen, Pingting Zhong, Bowen Liu, Mingguang He, Danli Shi

**Affiliations:** ^1^ School of Optometry The Hong Kong Polytechnic University Hong Kong SAR China; ^2^ Faculty of Science and Technology University of Macau Macau China; ^3^ Institute For Interdisciplinary Information Sciences Tsinghua University Beijing China; ^4^ Department of Mechanical Engineering The Hong Kong Polytechnic University Hong Kong SAR China; ^5^ Research Centre for SHARP Vision (RCSV) The Hong Kong Polytechnic University Hong Kong SAR China; ^6^ Centre for Eye and Vision Research (CEVR) Hong Kong SAR China

**Keywords:** clinical autonomy, embodied intelligence, human–robot collaboration, medical robotics, safety‐critical robotics, shared control

## Abstract

Robotic systems are entering clinical care, but full autonomy remains constrained by patient variability, safety requirements, dynamic clinical environments, and the need for professional oversight. Human–robot collaboration (HRC) offers a more realistic path: clinicians retain judgment and responsibility, while robots and AI support sensing, planning, action, and decision‐making within defined task boundaries. In this narrative review, we examine recent advances in clinical HRC from a systems perspective, covering system architecture, learning and control, safety mechanisms, autonomy assessment, and real‐world deployment. We propose a dual‐brain framework, comprising a professional brain for clinical reasoning and decision support, and a physical brain for embodied sensing, planning, and task execution. Using examples from imaging, rehabilitation, surgery, and outpatient care, we argue that clinical autonomy is developing in stages from clinician‐supervised imaging and decision support to bounded robotic subtasks and workflow‐level coordination, instead of through unsupervised replacement of healthcare professionals. We further discuss how robustness, trust, accountability, and human factors shape safe adoption in practice. By framing autonomy as collaborative rather than substitutive, this review provides a unifying foundation for designing and evaluating the next generation of autonomous clinical systems.

## Graphical abstract

This graphical abstract summarizes the conceptual structure of human–robot collaboration (HRC) for autonomous clinical systems. The framework is organized around a dual‐brain architecture in which a professional brain supports clinical reasoning tasks such as diagnosis, image analysis, treatment planning, and patient monitoring, while a physical brain enables sensing, data processing, task execution, and human–robot interaction. Multimodal data exchange links these two components to coordinate decision‐making and embodied action. Building on this architecture, a collaborative autonomy model illustrates how different levels of autonomy emerge from the interaction between human involvement and robot capability, ranging from assistant autonomy to task‐level and high‐level autonomy. Representative clinical applications demonstrate how these collaborative systems are deployed across healthcare environments, including patient intake robots, hospital navigation robots, robotic ultrasound systems, and surgical assistance robots. Together, the framework highlights how HRC enables safe, scalable, and clinically practical autonomy.

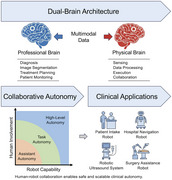

## 1 Introduction

Global healthcare systems are under growing stress from population aging, workforce shortages, and increasingly complex clinical decision‐making. These pressures have accelerated the use of robotic and artificial intelligence (AI) systems across tasks ranging from patient intake and imaging to rehabilitation, surgery, and long‐term monitoring. Medical automation has therefore moved beyond rule‐based assistance and teleoperation toward embodied, learning‐enabled systems that can sense the clinical environment, interpret human input, and support more complex tasks [1, 2, 3, 4, 5]. These developments may improve efficiency, consistency, and access to care, particularly in resource‐constrained settings [6, 7, 8].

Recent progress in medical robotics and clinical AI reflects a broader shift from isolated automation to clinician‐guided human–AI–robot collaboration. AI systems now assist with image analysis, workflow triage, and report drafting [9, 10]; surgical robots increasingly support anatomical recognition, risk warning, and constrained assistance [11, 12, 13]; and precision‐medicine tools are beginning to inform personalized risk stratification, treatment selection, and perioperative planning [14, 15, 16, 17]. Across these settings, autonomy is best understood as bounded and supervised, where clinicians remain responsible for interpretation, consent, escalation, and final clinical decisions. However, progress remains fragmented. High‐level clinical intelligence is rarely integrated with low‐level embodied execution, and evaluation often focuses on isolated technical metrics rather than system‐level autonomy, safety, workflow fit, and human factors.

Despite rapid technical advances, autonomous and semi‐autonomous robotic systems remain difficult to deploy in routine clinical care. Most systems still work in narrow settings, rely on fixed behaviors, or require close clinician control [18, 19, 20]. In safety‐critical environments where patients, workflows and ethical responsibilities vary continuously, full autonomy is rarely realistic. The central goal is therefore not to replace clinicians, but to build collaborative systems that can act independently when appropriate while remaining guided by human judgment, clinical accountability, and institutional safeguards [21, 22, 23].

Human–robot collaboration (HRC) offers a pragmatic paradigm for this goal by distributing sensing, reasoning, and control between humans and robots according to task demands, risk levels, and user expertise [24]. In clinical care, HRC connects human expertise with AI‐enabled robotic systems. Through shared control, adaptive interaction, and supervision, robots contribute precision, endurance, and rapid data processing, while humans retain authority over clinical reasoning, ethical decisions, and unexpected events [6, 25, 26, 27]. In this review, autonomy refers to the bounded, task‐specific ability of a human–AI–robot system to interpret clinical context, reason over available information, act or recommend actions, and escalate uncertainty under human authority and safety constraints. This framing connects robotic decision‐making with clinical reasoning: robots translate perception into action, whereas clinicians translate signs, images, and histories into treatment decisions through human judgment and multidisciplinary deliberation. Yet current studies remain scattered across disciplines, departments, and technical modules. A comprehensive review is therefore needed to connect clinical intelligence, embodied execution, human oversight, and safety evaluation within a unified framework for autonomous clinical systems.

**FIGURE 1:**
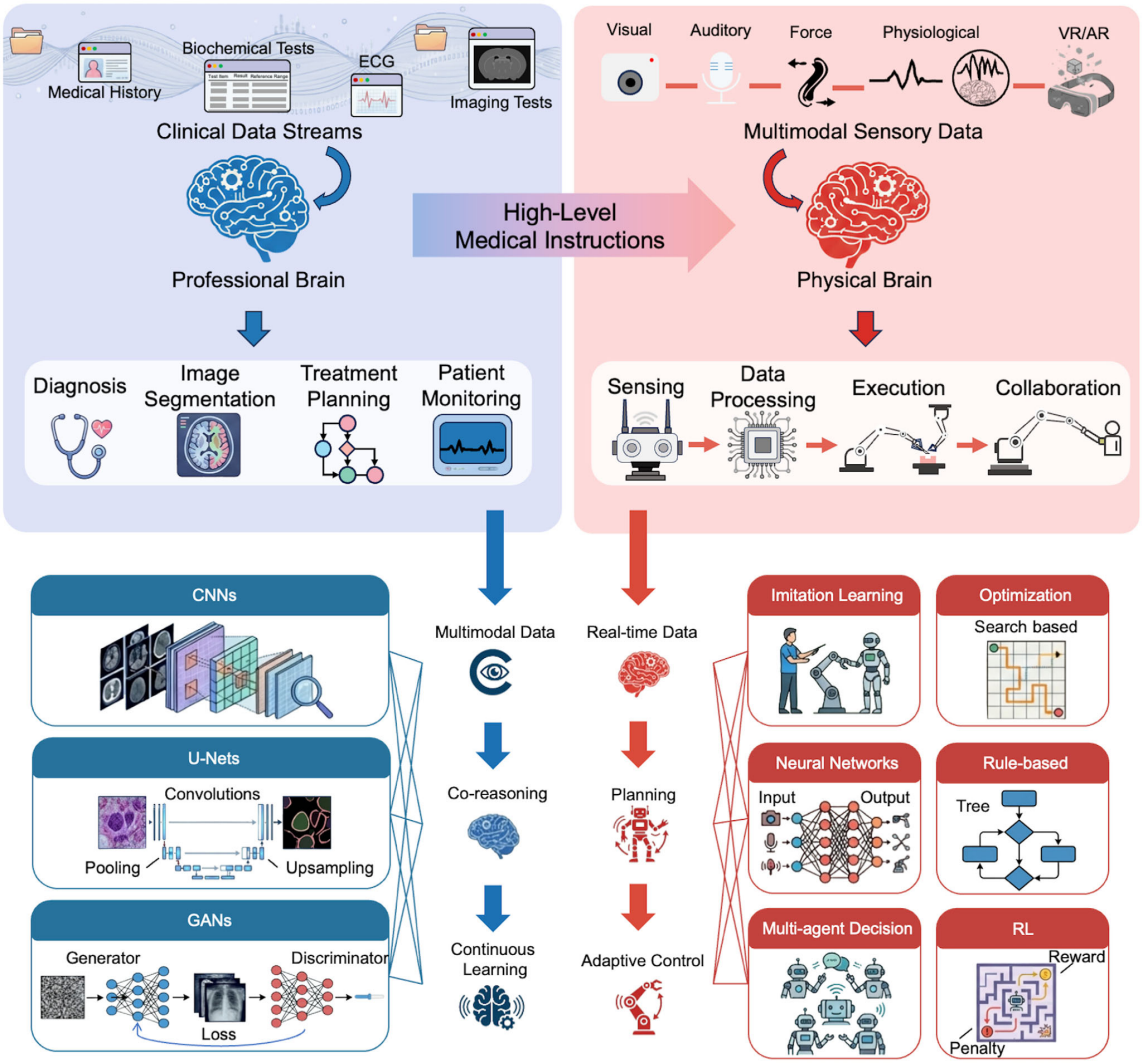
General Structure and Development of Clinical HRC Systems. Clinical HRC systems are organized around two complementary parts: the professional brain and the physical brain. The professional brain integrates clinical data streams to generate high‐level medical instructions, supporting diagnosis, image segmentation, treatment planning, and patient monitoring. These functions are enabled by data‐driven models and are characterized by multimodal integration, co‐reasoning, and continuous learning. The physical brain receives multimodal sensory inputs to enable sensing, data processing, task execution, and collaboration. Its decision‐making and control rely on a range of algorithms, allowing real‐time planning and adaptive control during clinical interaction. AR, augmented reality; CNN, convolutional neural network; ECG, electrocardiogram; GAN, generative adversarial network; RL, reinforcement learning; VR, virtual reality.

Here, we present a narrative, systems‐oriented review of clinical HRC. We identified representative literature through iterative searches of PubMed, Web of Science, IEEE Xplore, and Google Scholar using combinations of terms including medical robotics, human‐robot collaboration, shared control, autonomous surgery, robotic ultrasound, clinical AI, multimodal sensing, safe control, and clinical workflow automation. We prioritized studies involving robotic embodiment or clinically relevant collaborative tasks, human oversight or shared control, safety, validation, governance, and representative clinical application domains. Because the evidence spans proof‐of‐concept systems, preclinical validation, early clinical studies, and implementation reports, findings were synthesized qualitatively. We also propose a dual‐brain framework that separates two linked functions: a professional brain for clinical reasoning and decision support, and a physical brain for embodied sensing, planning, and task execution. This framework does not replace established perception‐planning‐control or shared‐control architectures. Instead, it makes explicit a clinically important distinction between reasoning about care and acting safely in the patient‐facing environment. Figure 1 shows the overall dual‐brain architecture and the interaction between professional and physical components. We examine the key technologies behind each component, including multimodal perception, context modeling, explainable reasoning, robotic sensing, planning, interaction, and safety control. We then use examples from imaging, rehabilitation, surgery, and outpatient care to show how different modes of collaboration produce different levels of autonomy. We further establish an evaluation framework for defining task boundaries and assessing autonomy, safety, workflow integration, and human factors. Finally, we argue that clinical HRC should integrate robotic capability into clinical workflows in a safe, accountable, and human‐centered way rather than maximize autonomy for its own sake.

## 2 Roles and Interaction Modalities in Clinical HRC

Clinical HRC depends on the integration of physical and cognitive collaboration in dynamic, unstructured healthcare environments. In these systems, humans and robots assume complementary roles that vary with task complexity, clinical risk, and user preference [28]. This section outlines these roles and the interaction modalities that support safe and effective collaboration.

### 2.1 Roles of Robotic Systems

In clinical HRC, the professional brain uses AI to support knowledge‐based medical reasoning [29, 30]. It integrates medical knowledge, clinical data, and reasoning capabilities to process multimodal information in real time and support diagnosis, treatment planning, and monitoring. Its core functions include knowledge retrieval, clinical reasoning, dynamic feedback, and adaptive learning. These capabilities can assist clinicians with diagnosis [31, 32, 33], medical image segmentation [34], disease prediction [35], treatment recommendations [36, 37], and patient monitoring [38], improving the efficiency and consistency of healthcare.

The physical brain governs embodied execution. It complements human collaborators through endurance, precision, and rapid multimodal data processing, including vision [39], audio [40], force [8, 26, 41], and biosignals such as electroencephalography (EEG) or electromyography (EMG) [42, 43]. By integrating environmental perception, motion planning, and compliant actuation, it translates high‐level medical instructions into spatially and dynamically feasible robotic actions [44, 45, 46]. Together, the professional and physical brains form a layered intelligence architecture in which clinical reasoning guides embodied control, supporting scalable, safe, and clinically meaningful autonomy.

### 2.2 Roles of Human Collaborators

In most HRC systems, clinicians remain the final decision‐makers and ethical gatekeepers [47]. They supervise robotic actions, provide high‐level instructions, and intervene when unexpected events occur. These instructions can be delivered through demonstrations, voice, or gestures, enabling more natural collaboration [48]. Timely human intervention helps protect patient safety, correct system errors, and preserve clinical and ethical accountability [25, 47]

Human collaborators do more than supervise. Clinicians define the task, choose the appropriate level of autonomy, judge whether sensor data are clinically meaningful, and decide whether a recommendation should be accepted, revised, or rejected. Other staff, including nurses, technicians, therapists, and caregivers, contribute essential workflow knowledge by positioning patients, preparing instruments, monitoring discomfort, and conveying contextual details that robots may miss [47]. Patients are also active collaborators: their consent, preferences, movement, language, and tolerance determine whether a technically feasible action is clinically appropriate [25].

Clinical HRC is therefore a model of distributed responsibility, not simple human command over a machine. The professional brain may summarize evidence, estimate risk, or suggest plans, while the physical brain may stabilize motion or perform bounded subtasks. However, humans retain authority over clinical values, priorities, and exceptions [49, 50]. This is especially important when autonomy is uncertain. Systems should clearly show their confidence, intended action, and fallback options so that users can calibrate trust and intervene when needed. Clear role allocation also improves documentation and training by making it explicit who initiated the task, approved escalation, performed rescue, and remained accountable for the final clinical decision [18, 51].

### 2.3 Interaction Modalities

Interaction modalities connect clinical intent with robot behavior and make robot status visible to human users. Effective interaction should be bidirectional: humans give goals, constraints, and corrections, while robots report their confidence, progress, and risk. In physically shared tasks, humans and robots may also share control. When uncertainty or risk increases, the system should support clear escalation, including slowing down, stopping, or handing control back to a human.

Human‐to‐robot communication can occur through voice, gesture, demonstration, and physiological signals. Voice enables natural, hands‐free control, which is especially useful during surgery or emergencies when clinicians cannot use their hands [40, 45, 52]. Gesture and demonstration can define spatial goals or teach task patterns, whereas EMG, EEG, electrocardiography (ECG), eye tracking, and related biosignals may indicate user intent, workload, fatigue, or stress [42, 43, 47, 48, 53]. These channels are most useful when they reduce interaction burden without weakening confirmation for high‐risk commands.

Robot‐to‐human feedback includes visual interfaces, confidence displays, explanations, alerts, and AR or VR overlays. Graphical interfaces can provide transparency, manual override, and visual feedback during surgical navigation or diagnostic assistance [54]. AR and VR can overlay guidance and spatial information directly into the clinician's field of view, supporting intraoperative assistance and remote care [55]. In safety‐critical settings, feedback should distinguish routine updates from events that require escalation, and explanations should be brief enough to support timely clinical decisions [56, 57, 58, 59].

Shared physical interaction is important when humans and robots work on the same patient, instrument, or workspace. Force feedback, haptics, compliant control, virtual fixtures, and co‐manipulation allow robots to stabilize motion or regulate contact while clinicians retain situational authority [60, 61]. In rehabilitation, robotic ultrasound, and microsurgery, these approaches help balance precision with patient comfort and clinician judgment.

Together, these modalities turn robotic capability into a usable clinical partnership. Good interaction design should prompt confirmation, slow or stop motion, trigger alerts, or transfer control back to clinicians when confidence falls, sensor data conflict, patient discomfort appears, or the task moves beyond its predefined boundary [62, 63, 64]. In this sense, interaction is not only a matter of usability but also a safety mechanism for preserving human oversight in autonomous clinics.

## 3 System Architecture and Implementation of HRC in Autonomous Clinics

Effective HRC depends on five core technological modules: sensing, planning, task execution, interaction, and safe control. Together, these modules allow robots to perceive clinical context, plan appropriate actions, execute tasks, communicate with human users, and remain within safe operating boundaries. This section describes each module and highlights its implications for clinical collaboration.

### 3.1 Professional Brain

The professional brain represents the cognitive layer of clinical HRC. It interprets complex medical information and converts it into useful guidance for robotic action. Three core technologies support this function: multimodal perception, context modeling, and clinical reasoning. To operate in dynamic and uncertain healthcare settings, the professional brain should integrate data across modalities, learn from clinical context and memory, and generate outputs that are safe, explainable, and clinically meaningful.

#### 3.1.1 Multimodal Perception

Multimodal perception allows the professional brain to integrate heterogeneous clinical data rather than relying on a single information source. Its development depends on high‐quality data collection, curation, and annotation. In medical imaging, commonly used modalities include magnetic resonance imaging (MRI), ultrasound, carotid ultrasound, and chest X‐rays, as well as ophthalmic imaging such as color fundus photography (CFP), optical coherence tomography (OCT), and fundus fluorescein angiography (FFA). Public datasets have accelerated progress by linking images with structured labels or clinical context. For instance, the MedMNIST database aggregates 10 public medical datasets, containing a total of 450,000 low‐resolution (28 × 28 pixel) medical images [62], whereas the BraTS dataset provides multimodal MRI images for brain tumor segmentation [65]. In ophthalmology, recent datasets increasingly integrate richer clinical semantics, including angiography reports and visual‐question‐answering (VQA)‐style benchmarks [66, 67].

Recent advances in vision‐language learning have further strengthened multimodal perception. Contrastive Language‐Image Pre‐training (CLIP) aligns textual and visual representations, enabling models to connect images with descriptions and clinical concepts [68]. Fine‐tuning and adaptation of such models have shown value in medical imaging tasks. Feature‐wise linear modulation offers another strategy for fusing image and text features by dynamically adjusting the representation space, improving performance in tasks such as segmentation and classification [69].

Physiological signals provide another important source of clinical context. ECG captures cardiac electrical activity, EMG reflects neuromuscular function, magnetoencephalography (MEG) can localize brain activity, and near‐infrared spectroscopy (NIRS) provides non‐invasive information on cerebral hemodynamics and oxygenation. Continuous signals such as photoplethysmography (PPG) and respiratory monitoring further support longitudinal assessment. By combining imaging, text, physiological signals, and clinical records, multimodal approaches can improve robustness and support more personalized diagnosis, risk assessment, and task planning.

Multimodal machine learning integrates various data streams to improve disease understanding and prediction [70]. For example, A2FSeg uses adaptive multimodal fusion for brain tumor segmentation and achieved strong performance on the BraTS2020 dataset [71]. In clinical HRC, such multimodal inputs provide the professional brain with the contextual evidence needed to guide diagnosis, estimate risk, and inform downstream robotic actions.

#### 3.1.2 Context Modeling

Context modeling allows the professional brain to interpret current information in relation to recent events, longitudinal records, and prior clinical experience. A useful system should combine short‐term memory, which captures recent environmental changes and interaction history, with long‐term memory, which stores accumulated knowledge from previous cases [72]. This combination helps the system adapt to dynamic clinical situations while maintaining continuity across visits, tasks, and decisions.

In medical image analysis, temporal and contextual modeling are commonly supported by recurrent and attention‐based architectures. Long short‐term memory (LSTM) networks can process sequential data, making them suitable for image series, video monitoring, and dynamic clinical signals. Combined convolutional neural network (CNN)‐LSTM models first extract image features and then model their temporal relationships. For example, Agrawal et al. used CNN‐derived features from segmented fundus ridge‐line images and LSTM‐based sequence modeling to classify stages of retinopathy of prematurity (ROP) [73]. Transformer models extend this capacity by using self‐attention to capture long‐range dependencies, making them useful for large‐scale imaging datasets, longitudinal records, and multimodal clinical histories [74, 75].

For clinical HRC, context modeling is important because robot actions rarely depend on a single observation. A scan‐quality warning, a patient movement, a prior diagnosis, and a current symptom may all influence whether a robot should continue, repeat a step, slow down, or request human review. Context‐aware modeling therefore provides the bridge between raw perception and clinically appropriate action.

#### 3.1.3 Clinical Reasoning

Clinical reasoning enables the professional brain to operate in complex and uncertain medical environments. It should combine data‐driven inference with explicit clinical constraints, producing outputs that are transparent, context‐aware, and suitable for human confirmation. Relevant inputs include imaging, clinical text, medical records, symptom assessments, patient feedback, and real‐time interaction data.

Reasoning systems may use knowledge graphs (KG), retrieval‐augmented generation, probabilistic models, and rule‐based safety layers to link symptoms, imaging findings, longitudinal records, guidelines, and patient‐specific risk factors [10, 76]. In this setting, the model should not simply produce a diagnosis or plan. It should show the evidence used, identify missing information, estimate uncertainty, and suggest the next action for clinician review. This is significant in autonomous clinics, where reasoning errors may directly affect robot behavior unless they are filtered through human oversight and safety controls.

The reasoning layer also connects the professional and physical brains. For example, an imaging model may detect a lesion, an interaction model may identify patient anxiety, and a planning model may decide whether the robot should repeat the scan, adjust contact force, pause, or request human review. Similar reasoning can support anatomical recognition, risk warning, perioperative planning, and individualized treatment selection in surgery and precision medicine, while leaving final decisions to the multidisciplinary team [13, 15, 16, 77]. To be clinically useful, these outputs should be calibrated, traceable, and task specific: a high‐confidence segmentation, a low‐confidence triage recommendation, and an uncertain motion plan should trigger different responses. Future systems should therefore report not only accuracy but also explanation quality, uncertainty calibration, failure recovery, and how often clinicians override or modify the proposed action [78, 79, 80, 81, 82].

Together, multimodal perception, context modeling, and clinical reasoning allow the professional brain to interpret complex medical information and operate in uncertain clinical environments. Its value depends not only on algorithmic performance, but also on robustness, explainability, and safe integration with human oversight and robotic execution.

### 3.2 Physical Brain

The physical brain enables embodied action. It first perceives the clinical environment and human activity through multimodal sensors, then converts this information into action through planning, execution, and safe control. This section reviews the sensing technologies used in medical robotics, the learning algorithms that support robotic decision‐making, and the control mechanisms required for safe and reliable operation.

#### 3.2.1 Sensing

Perception is the basis of situational awareness in clinical HRC. Physical sensors such as vision, audio, distance, and motion sensors can capture environmental dynamics, while biosignal and force sensors provide information about human physiology, intent, and contact. The visual system provides red‐green‐blue (RGB) and depth data for object recognition, gesture detection, motion tracking, and spatial mapping [83], while audio sensors capture speech and environmental sounds to support voice control and acoustic awareness [52]. Ranging and motion sensors, including light detection and ranging (LiDAR), millimeter‐wave radar, and inertial measurement units (IMUs), enable non‐contact mapping and movement tracking, supporting posture assessment, intent prediction, and rehabilitation monitoring [84]. Biological sensors can infer cognitive load and error perception through EEG, detect muscle activity and movement intention through EMG [42], and reflect stress or cognitive readiness through ECG, allowing robots to adapt their collaboration strategies [28]. Force and haptic sensors further support compliant contact, force regulation, and tactile feedback in procedures such as palpation or tissue manipulation [8, 26]. Common sensor types used in clinical HRC are summarized in Table 1.

**TABLE 1:** Commonly used sensors in clinical HRC.

Reference	Subcategory	Signal type	Task
**Vision**			
Ercolano et al. [87]	RGB camera	2D video frames (RGB)	Gesture recognition for robot‐led training in ASD therapy
Ghosh et al. [88]	RGB camera	Facial CNN	Deep learning‐based facial expression recognition to assess the intensity of patients’ pain‐related emotions.
Bussolan et al. [89]	RGB camera	Action units	Facial feature extraction for detecting the operator's mental workload during collaborative tasks.
Amprimo et al. [90]	RGB‐D camera	RGB and depth images	Validation of a depth‐enhanced vision framework for hand tracking in clinical rehabilitation.
**Audio**			
Abdollahi et al. [91]	Voice	Spoken dialogue and sentiment	A conversational agent for older adults that detects the user's emotional state.
Davila et al. [56]	Voice	Whisper ASR	Voice‐controlled interface for a surgical robot assistant to reduce the surgeon's cognitive load.
Ghosh et al. [88]	Spectrogram	Speech audio signals	Speech signal feature analysis to assess pain‐related emotions in an IoT‐based framework.
Garcia et al. [57]	Acoustic	Pepper mic	Multimodal emotion recognition framework that adapts to individual emotional expressions via fine‐tuning.
**Ranging/motion**			
Qian et al. [58]	LiDAR‐IMU fusion	LiDAR point cloud and IMU (six‐axis) data	Robust dynamic localization and 3D mapping in complex outpatient environments.
Kulhánek et al. [59]	mmWave radar	Radar signals	Patient presence detection using a bed‐mounted radar sensor, with 94% accuracy.
Shi et al. [60]	mmWave radar	Radar signals	Method for multi‐target vital sign monitoring and localization in hospital wards.
Li et al. [61]	mmWave radar + IMU	Radar and inertial data	Millimeter‐wave radar‐assisted SLAM system for localization and mapping in complex medical environments.
Shi et al. [92]	mmWave radar + IMU	Radar and inertial data	HAR including walking, running, and fall detection.
**Biological**			
Kim et al. [63]	sEMG	EMG signals (electrical potentials)	Gesture‐driven, contactless control of medical devices.
Cho et al. [64]	ECG	HRV	Assessment of cognitive load and stress during collaborative human–robot interactions.
Ding et al. [93]	EEG (motor imagery EEG)	Brain signals (μ‐rhythm EEG)	Mind‐controlled robotic hand enabling individual finger movements.
Leerskov et al. [94]	EEG‐based BCI	Movement‐related EEG signals (MRCP)	Intent detection to trigger FES and robotic limb exercise for early rehabilitation.
**Force**			
Li et al. [95]	Tactile	Pixel‐level elastic deformation images	Automated tissue palpation and tumor identification in robot‐assisted surgery.
Arshad et al. [96]	Tactile	Capacitive plate	Design of a uniaxial tactile sensor with a highly linear response for surgical force feedback.
Ouyang et al. [97]	Haptic	FlexiForce A201	Biomimetic tactile feedback system for tumor localization in Da Vinci robot‐assisted surgery.
Gong et al. [98]	Intent	Load sensors	Force‐sensor fusion algorithm for an intelligent rehabilitation robot to assist gait training in stroke patients.

Abbreviations: ASD, autism spectrum disorder; ASR, automatic speech recognition; BCI, brain‐computer interface; CNN, convolutional neural network; ECG, electrocardiography; EEG, electroencephalography; FES, functional electrical stimulation; HAR, human activity recognition; HRV, heart rate variability; IMU, inertial measurement unit; LiDAR, light detection and ranging; mmWave, millimeter‐wave; MRCP, movement‐related cortical potential; RGB, red‐green‐blue; RGB‐D, red‐green‐blue plus depth; sEMG, surface electromyography; SLAM, simultaneous localization and mapping.

Single‐sensor data are rarely sufficient for robust clinical perception, making multimodal fusion particularly important [85, 86]. For example, combining facial cues with vocal tone can improve emotional assessment in mental health applications [53], while integrating force sensing with visual tracking can improve the safety of physical assistance. These perceptual signals provide the basis for downstream decision‐making and task allocation. However, sensor noise, patient variability, and workflow integration remain major barriers, highlighting the need for robust multimodal fusion and validation in real clinical environments.

#### 3.2.2 Planning

Planning transforms perception into collaborative action [99]. It uses environmental feedback to select, refine, and adapt robotic behavior according to task demands and clinical constraints. Rule‐based systems offer transparency and predictability, but they have limited flexibility in unstructured environments. In contrast, reinforcement learning (RL) and multi‐agent frameworks improve adaptability under uncertainty [100, 101], while imitation learning (IL) enables robots to acquire expert‐like behavior from demonstrations [102]. Optimization algorithms are useful in well‐defined tasks, and neural networks can model complex state‐action relationships, although their decisions may be difficult to interpret. In practice, robotic decision‐making often combines several approaches. Hybrid IL‐RL methods can improve adaptability by fine‐tuning behavior cloning (BC) policies or using DAgger to reduce distribution shift. Additionally, self‐supervised and representation learning can extract richer state features, while world models support higher‐level prediction and planning. Together, these methods significantly strengthen the ability of robots to operate in complex and dynamic clinical environments (Table 2).

**TABLE 2:** Learning algorithms in the field of robotics.

Reference	Algorithm category	Key technology/representative algorithm	Key performance indicator
Prakash Gawas et al. [103]	Imitation learning	DAgger, stochastic expert models	Significantly reduce scheduling conflicts and enhance the stability of real‐time decision‐making.
Nazeer et al. [104]	Imitation learning	Soft DAgger, behavioral mapping	Provides a practical control solution to perform complex tasks in fewer samples with soft robots.
Duan et al. [105]	Imitation learning	Gaussian probabilistic imitation learning	The robot arm learns manifold trajectories in a polishing task.
Kim et al. [106]	Imitation learning	Behavioral cloning	Robots learning for surgical tasks involves mastering key operations, including tissue manipulation, needle handling, and knot tying.
Shi et al. [107]	Imitation learning	Behavioral cloning	The success rate of robot navigation increased by up to 25%, and improved by 4%–28% in real‐world bimanual manipulation tasks.
Komorowski et al. [108]	RL	FQI, AI clinician	The dosage of vasopressors and intravenous fluids for sepsis patients.
Taheri et al. [109]	RL	PPO, DDPG	Effectively avoiding collisions in complex environments by the robot.
Yand et al. [110]	RL	DQN	Improved the slow convergence and excessive randomness issues in robot path planning, enhancing the efficiency of multi‐robot path planning.
Churamani et al. [111]	RL	MCCNN, emotion‐driven RL	Improve the success rate of emotional game interactions and enhance social naturalness for human robots.
Husaković et al. [112]	RL	SAC, CQL	100% success rate in simulation and 80% task success rate in real‐world operation.
Ma et al. [113]	RL	TRPO, predictive reward modeling	Achieve smoother trajectories for the robot and effectively improve anatomical accuracy.
Kuo et al. [114]	RL	GANs	The robot can autonomously identify its environment and conduct self‐training.
Melo et al. [115]	RL	PPO	The robot learns complex actions in a short period of time.
Chow et al. [116]	RL	DDPG, PPO	Continuous action optimization for indoor robot navigation.
Zhu et al. [117]	Evolutionary learning	PPO	Achieved self‐evolution of bipedal wheeled robots for movement in complex terrains.
Pagliuca et al. [118]	Evolutionary learning	CMA‐ES, xNES, OpenAI‐ES	OpenAI‐ES achieves better performance for protecting groups against predators or speeding up the foraging process.
Miki et al. [119]	Multi‐agent decision algorithms	Decision‐making	Multi‐agent collaborative exploration and task selection.
Jolly et al. [120]	Multi‐agent decision algorithms	ANN	Achieved the advancement of intelligent decision‐making in multi‐agent systems.

Abbreviations: AI: artificial intelligence; ANN: artificial neural network; CMA‐ES: covariance matrix adaptation evolution strategy; CQL: conservative Q‐learning; DDPG: deep deterministic policy gradient; DQN: deep Q‐network; FQI: fitted Q‐iteration; GANs: generative adversarial networks; MCCNN: multi‐channel convolutional neural network; PPO: proximal policy optimization; RL: reinforcement learning; SAC: soft actor‐critic; TRPO: trust region policy optimization; xNES: cross‐entropy method for natural evolution strategies.

#### 3.2.3 Task Execution

Task execution translates high‐level planning into precise physical operations. Shared‐control architectures allow clinicians to retain authority while benefiting from robotic precision, stability, and endurance [121, 122]. Force feedback, compliant control, and disturbance rejection can further improve accuracy and safety, especially in microsurgery [96, 123]. Recent developments in IL and hierarchical transformer models, such as the Surgical Robot Transformer, have enabled autonomous completion of surgical subtasks [106]. The vision‐language‐action models further integrate perception, reasoning, and control, allowing robots to adjust their actions in real time during task execution [124]. These developments support increasing task‐based autonomy while preserving the need for clinical supervision.

#### 3.2.4 Human–Robot Interaction

Language, gesture, visual feedback, and physiological signals form multimodal communication loops between humans and robots. Natural language processing (NLP) and large language models (LLMs) can support contextual dialogue and cooperative reasoning in clinical settings [124, 125]. KG improve the contextual understanding of clinical relationships [126], sentiment analysis models help interpret user emotional states [88], and intent recognition models (IRM) identify user goals and commands [127]. Safe cooperation also requires clinicians to understand robotic states, intention, and limitations. Explainable AI, visual interfaces, and multimodal feedback can provide transparent information about confidence, progress, and execution status [128, 129]. Together, these interaction mechanisms support closed‐loop, trustworthy collaboration in autonomous clinical systems.

#### 3.2.5 Safe Control

Safety is especially important in clinical HRC because robots often operate near patients and clinicians in dynamic environments. Safe control forms the final layer of the autonomous‐clinic loop, combining multi‐level monitoring with human‐in‐the‐loop supervision. Robust control mechanisms are needed to manage uncertainty, reduce risks, and ensure that human authority remains central in clinical decision‐making.

*Precision control*: High‐precision motion control is fundamental for manipulating delicate instruments, particularly in fields such as ophthalmology, where minor errors may cause serious tissue damage. Xiang et al. proposed an LSTM‐based force estimation method that achieved compliant control and high‐precision force tracking in continuum robots. Their approach improved safety in procedures such as radiofrequency ablation and swab sampling by maintaining precise and stable contact [130].

*Uncertainty management*: Safe clinical operation requires robots to account for sensor noise [131], environmental variation [132], and human behavior [133]. Modeling these uncertainties allows systems to anticipate failures, adopt conservative strategies, or defer to human oversight when needed [134, 135]. Real‐time monitoring and transparent supervision further enable timely human intervention during unexpected or high‐risk events [25, 47].

*Learning‐based safety certification*: Beyond empirical robustness, formal safety guarantees are increasingly being explored through learned certificates. Neural Lyapunov, barrier, and contraction functions enable data‐driven verification of stability, reachability, and constraint satisfaction in nonlinear robotic systems [136]. These methods can define mathematically grounded safety envelopes around learned controllers, offering a promising route toward certifiable autonomy in safety‐critical settings. Integrating such certificates with clinical task models may support verifiable, learning‐enabled medical robotics.

*Novel safety strategies*: Causal reasoning can help systems infer the causes of failures and detect unreliable AI outputs, including hallucinations from LLMs or image‐analysis models [51]. Other control strategies, such as shear‐thickening fluid controllers, can improve impact resistance while maintaining compliance, offering protection against unexpected disturbances [41]. Together, these approaches help keep robotic behavior within acceptable risk boundaries while preserving human authority.

Together, sensing, planning, execution, interaction, and safe control form the closed loop of the physical brain. Multimodal sensors capture the environment and human signals; planning algorithms translate perception into action; execution modules perform physical tasks; interaction channels maintain communication with clinicians and patients; and safe control provides the final safeguard through precise motion, risk monitoring, and timely human intervention.

The professional brain and physical brain together define the system‐level architecture of clinical HRC. The professional brain interprets multimodal clinical information and generates explainable guidance, whereas the physical brain translates this guidance into sensing, planning, execution, interaction, and safe control. Their integration enables clinically meaningful collaboration, but not unrestricted autonomy. The appropriate autonomy level depends on task scope, clinical risk, human authority, and the availability of reliable escalation and takeover mechanisms. The next section applies this architecture to define practical autonomy levels and representative clinical applications.

## 4 Autonomy Levels and Clinical Applications

This section connects the definition of autonomy with clinical practice. It first distinguishes autonomy from related concepts, including AI decision support, robot capability, workflow orchestration, and human authority. It then applies this framework to communication, imaging, rehabilitation, surgery, and cross‐departmental workflows.

### 4.1 Autonomy Level Rubric

Clinical autonomy should be reported across four linked dimensions: what the robot can sense, plan or execute; what the AI can recommend, prioritize or explain; how far the workflow can proceed without continuous input; and who sets goals, approves high‐risk actions, overrides the system and remains accountable. This approach prevents a single autonomy label from hiding important differences in decision support, motion execution, workflow progression, and human oversight [18, 19].

**FIGURE 2:**
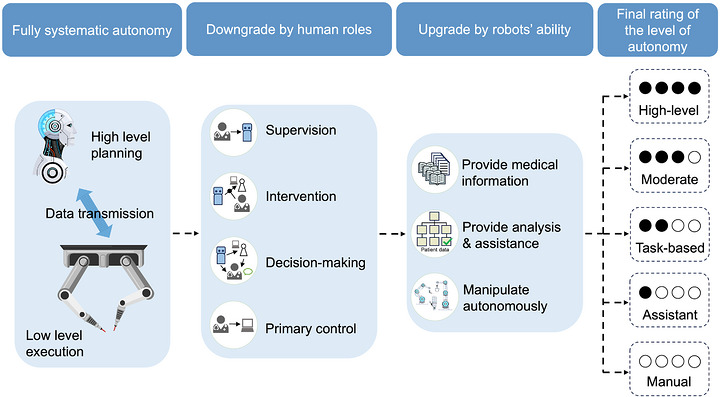
Overview of the approach to assessing the levels of autonomy. This framework characterizes autonomy as the combined outcome of human roles and robotic capabilities within a clinical HRC system. Autonomy is interpreted as a balance between system capability and human involvement: greater human intervention lowers the autonomy level, whereas greater robotic functional competence raises it.

HRC systems lie along a spectrum of autonomy shaped by human authority and robotic capability. Human involvement decreases as clinicians move from direct control to active decision‐making, intermittent intervention, and supervisory oversight. In parallel, robotic autonomy increases as systems move from displaying information to providing analytic support, assisting bounded tasks, and executing defined actions under safety constraints [137]. As shown in Figure 2, autonomy can be classified as low, assistant, task‐based, or high, depending on who holds decision authority, what the system can do independently, and when escalation, automatic stop, or human takeover is required. Table 3 provides a practical rubric for reporting these levels. Because acceptable latency, force, diagnostic agreement, and intervention thresholds vary across imaging, rehabilitation, surgery, and outpatient care, autonomy should be assessed by task or module using context‐specific endpoints rather than fixed numerical cutoffs.

**TABLE 3:** Operational criteria and validation endpoints for autonomy levels in clinical human–robot collaboration.

Level	Human authority	Robot/AI capability	Operational criteria	Validation endpoints	Typical examples
Low autonomy	Continuous human control	Stabilization, filtering, display, and mechanical augmentation	No independent clinical task completion and immediate override available	Latency, mechanical safety, ergonomics, and failure‐safe behavior	Tremor compensation and passive instrument stabilization [138, 139], navigation display [140], and haptic feedback rendering [141]
Assistant autonomy	Human approves recommendations or motions	Guidance, warning, segmentation, and constrained assistance	Narrow function and human confirmation required before consequential action	Diagnostic agreement, false‐alarm rate, explainability, and workload reduction	AI image pre‐screening and workflow triage [17], surgical trajectory suggestion [142], and voice‐controlled assistant [143]
Task‐based autonomy	Human sets goals and supervises	Bounded sensing, planning, and execution	Defined task boundary, escalation, or automatic stop when confidence is low	Task success, intervention rate, safety‐stop rate, force/trajectory error, and sim‐to‐real robustness	Robotic ultrasound scanning [144, 145], autonomous suturing subtask [146, 147], sim‐to‐real surgical skill transfer [148], and adaptive rehabilitation exercise [149, 150]
High autonomy	Policy‐level authorization and exception audit	Multi‐step workflow orchestration	Multi‐step workflow, rare intervention, audit trail, and governance controls	Clinical outcomes, exception rate, escalation appropriateness, auditability, bias monitoring, and user acceptance	Structured intake and triage [151] and long‐term companion monitoring [152]

**FIGURE 3:**
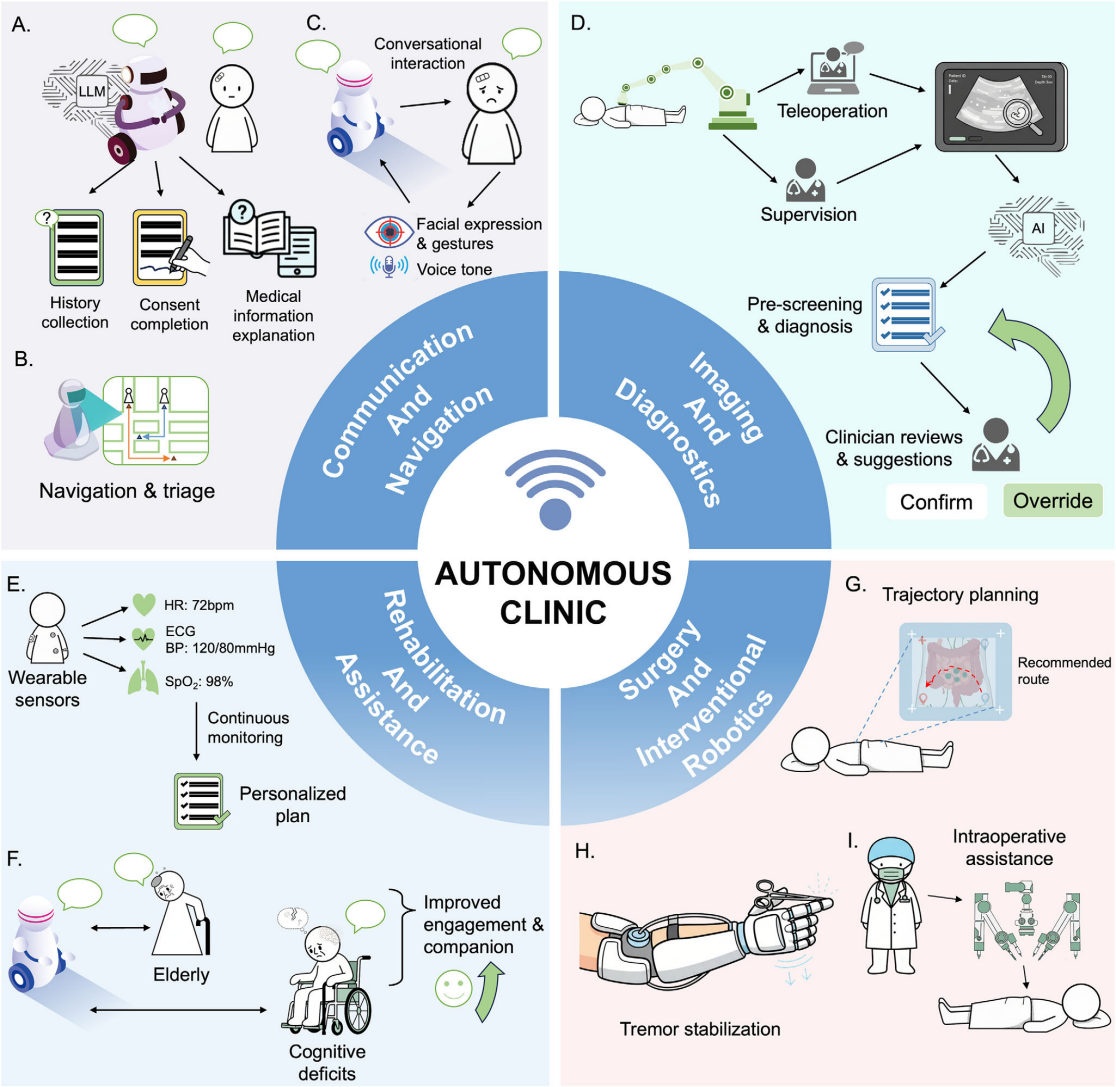
Use cases of clinical HRC systems. (A) **Task‐based to high‐level autonomy**. LLM‐based intake robots conduct history collection and consent completion with minimal human oversight. (B) **Task‐based autonomy**. Mobile robots perform navigation and triage, providing routing decisions based on real‐time patient flow. (C) **Assistant‐level autonomy**. Emotion‐aware social robots support conversational engagement in mental health contexts. (D) **Assistant to task‐based autonomy**. Robotic ultrasound systems enable supervised scanning, while AI performs pre‐screening and diagnostic suggestions. (E) **Task‐based autonomy**. Wearable‐sensor monitoring generates adaptive care plans under clinician supervision. (F) **High‐level autonomy**. Companion robots deliver cognitive and social support for long‐term rehabilitation. (G) **Assistant‐level autonomy**. AI‐driven trajectory planning suggests optimal interventional routes for clinician review. (H) **Assistant‐level autonomy**. Robotic tremor compensation stabilizes fine motor tasks during microsurgery. (I) **Low‐level autonomy**. Robots augment intraoperative manipulation and precision while surgeons retain primary control.

In clinical practice, HRC is being explored across the full care pathway, from routine patient interactions to specialized surgical procedures. Applications now include triage, imaging, surgical assistance, decision support, rehabilitation, and multi‐departmental integration. As systems mature, cross‐departmental coordination will become increasingly important for achieving higher autonomy while maintaining clinical safety and trust. Appropriate autonomy design therefore requires balancing task complexity, clinical risk, and user expertise, so that robotic assistance supports rather than replaces human clinical judgment (Figure 3).

### 4.2 Communication and Navigation

Communication and navigation systems usually operate at task‐based to high autonomy. They can collect information, manage simple interactions, and support path planning, while clinicians retain responsibility for supervision and escalation. In outpatient and front‐line care, HRC can support patient intake, triage, and navigation. During triage, AI‐enabled robots can analyze symptoms and vital signs to recommend clinical routing, while mobile platforms can guide patients to examination rooms and adapt paths according to real‐time congestion or schedule changes [153]. These systems mainly operate under task‐based autonomy with clinician oversight to ensure safety in real‐world interactions. Intake robots powered by NLP and LLMs can assist with identity verification, medical history collection, and consent form completion through interviews or touchscreen interfaces. Specialized AI agents are also emerging for primary care consultations, providing a basis for future integration into robotic platforms [154].

In mental health, social robots with multimodal emotion recognition can interpret voice, facial expression, and body language to provide empathetic and context‐appropriate responses [52, 53]. Unlike conventional AI chatbots, these systems interact within physical care settings and therefore require clear clinician supervision for ethical and safe patient engagement.

### 4.3 Imaging and Diagnostics

Imaging and diagnostics generally span assistant to task‐based autonomy, because AI systems support interpretation while robots perform bounded acquisition or positioning tasks. Imaging is one of the most mature domains of clinical HRC. Robotic ultrasound systems can assist with probe positioning, force control, and image optimization, improving consistency and reducing operator fatigue, particularly in repetitive scans or remote deployment [49, 155, 156, 157]. In these shared‐control systems, clinicians provide high‐level guidance while robots refine motion and pressure [8, 26]. Recent work using RL and IL further shows that robots can learn expert‐level scanning trajectories from visual input [144].

On the diagnostic side, AI algorithms can pre‐screen images, label suspicious lesions, and propose differential diagnoses, while clinicians validate their outputs within a collaborative diagnostic loop [158, 159, 160]. Transparent reasoning and real‐time explanations may improve trust and efficiency, while also supporting realistic training simulations for medical education [143]. Similar frameworks are emerging in ophthalmology, where robotic systems support patient alignment and multimodal imaging, and AI agents interpret ocular data to identify pathologies [140, 161, 162, 163]. By combining imaging and decision support, these systems can improve diagnostic accuracy, efficiency, and standardization.

In imaging, AI is most valuable as workflow support rather than fully autonomous diagnosis. Current systems can prioritize time‐sensitive findings, segment anatomical structures, quantify lesions, and draft reports. However, these outputs become clinically meaningful only when radiologists and other specialists can review uncertainty, correct errors, and decide whether a repeat scan or additional assessment is needed [16, 81]. This human‐in‐the‐loop model is especially important because real‐world performance can vary across institutions, patient populations, image quality, and baseline workflow maturity.

### 4.4 Rehabilitation and Assistance

Rehabilitation systems typically operate at task‐based autonomy. Robots can execute adaptive, patient‐specific motor or cognitive tasks, but therapy plans and safety oversight remain human‐led. Rehabilitation and assistance involve long‐term patient interaction, where HRC systems support physical recovery and cognitive maintenance through adaptive engagement. Robotic rehabilitation devices can guide motor tasks while adjusting resistance or assistance based on real‐time physiological feedback, improving precision and reducing therapist workload [41, 164, 165]. Wearable and remote sensors extend monitoring beyond clinics and enable continuous home‐based care [43, 166]. Beyond physical recovery, social robots can promote engagement through reminders, conversation, and cognitive interaction, particularly for patients with early‐stage dementia, stroke, or chronic conditions [53, 167]. These adaptive systems may extend care to home‐based and remotely supervised settings, but large‐scale deployment remains limited by patient adherence, long‐term usability, and cost‐effectiveness.

### 4.5 Surgery and Interventional Robotics

Surgical HRC usually remains at low to assistant autonomy. Robots enhance precision, stability, and instrument handling, but surgeons preserve primary authority for planning, intraoperative decisions, and emergency takeover. Shared‐control systems such as the Da Vinci platform [168] and research systems like OR‐Pilot [55] improve dexterity and safety while keeping the surgeon in control. Computer vision can support tool handover and visual guidance [83], while exoskeletons can reduce arm tremor during minimally invasive surgery [169]. In endoscopic and vascular interventions, robotic systems may automate guidewire or catheter manipulation, while clinicians retain responsibility for trajectory planning and risk management [170]. In ophthalmic microsurgery, robots can improve precision through surgeon‐guided shared control [171]. Applications include stabilizing instruments against physiological tremor [138, 172], assisting in retinal or subretinal intervention [173, 174], and supporting cataract surgery [175]. Recent studies have demonstrated that multisensor fusion and multiview perception intraocular microsurgery can support accurate retinal and vascular injections with reduced positioning errors. These systems show how assistant autonomy may improve procedural consistency while preserving human authority [176]. Simulation‐based platforms provide force feedback and stereoscopic visualization for training [177], and RL in simulation can support pretraining of surgical policies before real‐world deployment [178]. Mixed reality can further augment intraoperative perception by integrating preoperative imaging, live video, and robot‐state feedback [55].

Current evidence calls for a cautious view of surgical autonomy. Most deployed surgical robots still operate at low autonomy levels, while emerging AI functions mainly support perception, workflow understanding, anatomical localization, trajectory planning, and risk warning [19, 77, 179]. Preclinical systems, such as autonomous suturing and simulation‐trained surgical policies, show that bounded robotic subtasks are feasible. In clinical interventional platforms, however, the main benefits remain improved precision, standardization, radiation reduction, and access, with clinicians retaining takeover authority [180, 181]. AI may also support early diagnosis, perioperative planning, intraoperative guidance, and precision medicine in gastric cancer surgery, but only when algorithmic outputs remain connected to surgical judgment, tumor heterogeneity, and multidisciplinary decision‐making [13].

### 4.6 Cross‐Departmental Integration and System‐level Collaboration

Higher autonomy requires integration across clinical departments. Modular architectures, standardized task representations, and robust planning frameworks are needed for coordinated workflows. Collaborative crash‐cart robots in emergency settings represent early progress toward integrated, high‐stakes systems [6, 7], while ethnographic studies show that real‐world deployment can reshape clinical routines, teamwork norms, and staff responsibilities [182]. Viewing robots as agents of organizational change is therefore significant in achieving sustainable system‐level autonomy.

### 4.7 Preclinical and Clinical Validation Evidence

The preceding sections describe where clinical HRC is being used; this subsection considers how evidence should be generated before wider clinical scaling. Validation should be staged and domain‐specific, rather than based on a single model of evidence. In imaging and robotic ultrasound, systems can move from simulation, phantom studies, or controlled scanning tasks to supervised comparative studies, because clinicians can review image quality, repeat acquisition, and interpret outputs before clinical action is taken [16, 145, 183, 184].

Rehabilitation and assistive robotics require a different translational pathway. Early studies should focus on feasibility, comfort, adherence, therapist‐adjustable assistance, and functional recovery beyond independent task completion. The goal is adaptive support under professional supervision, with patients remaining active participants in the care loop [149, 150, 185, 186].

Surgical and interventional HRC requires the most cautious path to translation, because physical errors can cause irreversible harm. Evidence should clearly distinguish simulation and phantom tests, animal or in vivo studies, first‐in‐human feasibility studies, and comparative clinical evaluation. Examples such as autonomous intestinal anastomosis, robotic‐assisted endovascular aortic repair, and robot‐assisted subretinal delivery illustrate how step‐level automation can be tested while preserving surgeon or interventionalist takeover authority [174, 180, 187].

Outpatient, nursing, and social robots need a workflow‐centered evidence model. Their main risks are less about physical injury and more about communication failure, inappropriate escalation, privacy, bias, and disruption of staff routines. Feasibility and implementation studies should therefore report exception handling, escalation appropriateness, staff workload, patient comfort, auditability, and user acceptance. Table 4 summarizes representative validation evidence across these pathways and collaborative mechanisms.

**TABLE 4:** Representative preclinical and clinical validation evidence across clinical HRC pathways.

Publication	Trial registration	Study status and phase	Objective	Human role	Autonomy level	Preliminary findings
**Robotic ultrasound and imaging**						
Su et al. [145]	NA	Published clinical validation; phase not applicable	Fully autonomous thyroid ultrasound scanning and nodule characterization	Safety staff supervise; clinicians compare and interpret findings	Task‐based autonomy	Human‐participant testing showed scan quality close to clinician‐performed manual thyroid ultrasound and supported thyroid nodule detection/ACR TI‐RADS feature extraction.
Liang et al. [183] and Zhang et al. [184]	NA	Published prospective comparative clinical studies; phase not applicable	5G telerobotic abdominal ultrasound for rural access	Remote sonologist or radiologist leads; on‐site assistant supports positioning and safety	Low autonomy	5G telerobotic abdominal ultrasound was feasible and safe, with comparable image quality/diagnostic consistency but longer examination time than conventional on‐site ultrasound.
NA	NCT06621823	Registered diagnostic trial; recruiting; phase not applicable; 160 estimated participants	R‐LYNUS robotic intraoperative ultrasound for gynecologic lymph‐node assessment	Surgeon and clinical staff supervise robotic intraoperative ultrasound; clinicians confirm diagnostic decisions	Low to assistant autonomy	No peer‐reviewed or registry‐posted outcome results were publicly available at the time of review.
**Rehabilitation and assistive robotics**						
Xie et al. [185]	ChiCTR2400083917	Published randomized clinical trial; ChiCTR record identified, WHO ICTRP status pending; phase not applicable	Bilateral soft‐exoskeleton gait training for subacute stroke	Therapist sets the training plan and supervises; patient actively participates in gait training	Low to assistant autonomy	After 20 sessions, soft‐exoskeleton training produced greater short‐term gains in walking independence, lower limb motor function, balance, gait speed, and gait symmetry than conventional treadmill training, with no serious adverse events.
**Surgical/interventional robotics**						
Saeidi et al. [187]	NA	Preclinical in vivo porcine validation; phase not applicable	STAR autonomous intestinal anastomosis	Surgeon selects or confirms plan, supervises execution, and can intervene	Task‐based autonomy	In phantom and porcine models, autonomous STAR improved suture consistency/accuracy versus manual or robot‐assisted benchmarks and showed favorable lumen patency/leak‐pressure measures.
Liang et al. [180] and Song et al. [181]	ChiCTR2400082087	Published first‐in‐human single‐arm study with in vitro validation; phase not applicable	Automatic robotic‐assisted endovascular aortic repair	Surgeon supervises console execution and defines takeover threshold	Task‐based autonomy	Automatic robotic EVAR achieved 100% technical and clinical success in four abdominal aortic aneurysm patients without major adverse events, after in vitro testing showed millimeter‐level precision and reduced fluoroscopy/radiation versus manual robotic control.
Weisz et al. [188]	NCT01275092	Completed Phase 2 trial; 164 participants	Robot‐assisted PCI using the CorPath 200 system	Interventional cardiologist controls the workstation and retains procedural decision‐making, with manual bailout available	Low to assistant autonomy	Clinical procedural success was 97.6%, device technical success was 98.8%, no device‐related complications occurred, and primary‐operator radiation exposure was reduced by 95.2%.
**Ophthalmic microsurgery**						
Kapetanovic et al. [174]	NCT03052881	First‐in‐human randomized clinical trial; completed; phase not applicable	Robot‐assisted subretinal drug delivery	Retinal surgeon telemanipulates and makes all clinical decisions	Low to assistant autonomy	In 12 participants, robot‐assisted subretinal tPA delivery was well tolerated and safely completed, with similar procedure times, retinal microtrauma, and short‐term visual acuity gains versus manual surgery.
**Outpatient, nursing, and social robots**						
Mlakar et al. [189]	ISRCTN96689284	Published randomized external pilot trial; ISRCTN status no longer recruiting/completed; phase not applicable	Socially assistive ward robot for education, support, and basic triage	Clinical staff define scripts, supervise escalation, and retain care responsibility	Assistant autonomy	In 229 surgical patients, SAR implementation was feasible with high retention and no substantial negative effects, but primary effects on engagement and perceived care quality were limited.

*Note*: Publications and trial registration numbers are listed separately. NA indicates that no corresponding publication or trial registration was identified for that field from public sources. For published studies, publication was considered to indicate completed or reported evidence status. Recruitment status, trial phase, and enrollment information are provided where publicly available.

Across these domains, evidence should be judged in relation to the intended clinical pathway [137]. A phantom or simulation study may be sufficient for early algorithm comparison, but not for patient‐facing claims. An animal study can support tissue‐contact safety, but may not capture workflow burden. An early clinical feasibility study can reveal usability and escalation problems before definitive efficacy testing. At every stage, studies should clearly define the human role, task boundary, and safety endpoint.

This staged approach is also important for understanding failure [82]. In low‐risk workflows, a temporary pause or repeat acquisition may be acceptable; yet, the same event may require immediate handover in surgery or invasive intervention. Validation should therefore report not only average performance but also system behavior under uncertainty, such as poor perception, abnormal contact force, ambiguous communication, and unexpected patient movement. This makes safety claims easier to interpret in real clinical terms.

Overall, clinical autonomy is graded and task specific. It depends on how human authority, AI reasoning, and robotic execution are combined for a given task. Communication, imaging, rehabilitation, surgery, and cross‐department workflow each require different balances between human control, decision support, bounded task execution, and workflow orchestration. The same technical capability may carry very different risk depending on invasiveness, patient vulnerability, workflow urgency, and whether human takeover is feasible. Autonomy should therefore be assigned and validated at the task level, with clear reporting of the human role, robot capability, escalation pathway, and evidence stage [18, 19, 78, 79]. Together, the taxonomy, application examples, and validation table provide a practical framework for comparing current systems and guiding future autonomous‐clinic development.

## 5 Evaluation Frameworks

Evaluating HRC in clinical environments requires a multidimensional framework that goes beyond robot‐centered performance. Given the inherently cooperative nature of HRC, evaluation should capture team performance across task execution, clinical outcomes, workflow integration, user experience, safety, explainability, and ethical accountability at different autonomy levels. Comprehensive evaluation is therefore important for validating current systems and guiding future clinical translation [8, 39].

Evaluation should also test the interface between the professional and physical brains, where many clinical failures may occur [190]. For example, an accurate diagnostic model can still create risk if its uncertainty is not communicated before a robot acts, and a precise controller can still be unsafe if the clinical task boundary is unclear. Evaluation should therefore follow the full loop from data acquisition and reasoning to action, feedback, escalation, and documentation [191]. This loop‐based approach reduces overreliance on isolated module‐level metrics and makes evaluation more relevant to autonomous clinics.

### 5.1 Evaluation of Professional Brain

The professional brain should be evaluated against its clinical goals: supporting safer decision‐making, reducing clinician workload, improving access, and enabling more personalized care. Its assessment should therefore focus on whether the system can interpret multimodal clinical information, generate reliable recommendations, communicate uncertainty, and trigger escalation when its confidence or task boundary is exceeded. In autonomous‐clinic systems, these outputs should also be interpreted in relation to how appropriately they guide downstream robotic action.

#### 5.1.1 Clinical Reasoning Performance

Evaluation of the professional brain primarily concerns the reliability and accuracy of clinical reasoning tasks, especially medical image analysis and multimodal decision support. Assessment should go beyond whether a task is completed to examine whether the system uses relevant inputs, handles incomplete or variable data, maintains consistency across time, and updates its recommendations as new information becomes available. These aspects are particularly important in complex workflows that involve multimodal inputs and multitask reasoning [190]. For example, in CT or MRI analysis, evaluation should consider whether the system accurately identifies lesions, preserves image quality, adapts to incomplete or variable data, and processes multi‐dimensional time‐series information efficiently [192, 193, 194, 195, 196]. Clinical texts and patient‐record processing should also be assessed. The system should extract relevant information from medical histories, assessment records and other clinical documents so that decision support reflects the actual conditions of the patients. Evaluation may therefore include symptom recognition, severity assessment, format robustness, and integration across heterogeneous data sources [197, 198].

#### 5.1.2 Quality of Outcomes and Benchmarking Against Baselines

Outcome evaluation should determine whether the professional brain produces clinically meaningful outputs rather than only technically correct predictions. In medical image analysis, relevant outcomes may include image clarity, diagnostic value, reproducibility, and segmentation accuracy [199, 200]. Agreement between system outputs and expert clinical diagnoses is a key indicator of clinical relevance [201]. Consistency between imaging‐based and text‐based diagnoses can further support trust in system performance. The agreement between predicted disease severity and subsequent clinical course is also important for assessing prognostic value [202, 203]. For personalized care, evaluation should include the relevance and precision of medical recommendations, as well as patient satisfaction and adherence [204, 205].

These measures help determine whether the professional brain provides advice that is accurate, explainable, and acceptable within clinical practice. Together, reasoning performance and outcome quality provide a practical framework for evaluating the clinical effectiveness of the professional brain.

### 5.2 Evaluation of Physical Brain

Unlike the professional brain, the physical brain acts directly in the clinical environment and may interact with patients, clinicians or instruments. Its evaluation should therefore emphasize embodied performance: whether robotic actions are accurate, timely, compliant with safety constraints, and responsive to human input. In this context, technical success should be interpreted together with physical safety, workflow fit, and the consequences of failed or delayed action.

#### 5.2.1 Task Execution Performance

For the physical brain, task performance directly concerns the robot's reliability and accuracy in autonomous and collaborative actions. Assessment should extend beyond completion rates to include temporal alignment, adaptability, and workflow continuity. In applications such as ultrasound scanning or diagnostic navigation, evaluation should consider target localization, image quality, and the system's ability to adjust when clinicians or patients move unexpectedly [170, 206].

Because clinical environments are dynamic and safety‐critical, evaluation cannot rely solely on in situ testing. Simulation‐based evaluation is important for stress‐testing learned policies in high‐fidelity virtual environments before clinical translation. Recent advances in world models and foundation‐model‐driven simulators allow repeatable testing under controlled perturbations and edge cases. Systems such as Cosmos‐Surg‐dVRK illustrate how surgical robot policies can be evaluated online in simulation [207]. Simulation‐based benchmarking should be paired with structured sim‐to‐real validation to confirm that safety margins and performance metrics are preserved after deployment.

In both simulated and real clinical settings, physical‐brain evaluation should report not only aggregate success rates but also failure modes, safety‐stop events, near‐miss events, and intervention frequency. Collaborative contexts additionally require measures of workflow fluency, interruption recovery, and responsiveness to real‐time commands. Robustness to clinical variability and unpredictable events remains a key benchmark for practical deployment [84].

#### 5.2.2 Quality of Outcomes and Benchmarking Against Baselines

Beyond functional execution, HRC evaluation should assess whether robotic action improves clinical outcomes. Metrics differ by domain. Image‐guided interventions may focus on image clarity, diagnostic value, and reproducibility [8, 26, 39]; rehabilitation and surgery contexts may emphasize motion accuracy, functional recovery, or muscle activation [187, 208]; cognitive or conversational agents can be assessed through triage accuracy and the appropriateness of verbal and nonverbal responses [53, 209, 210].

Quantitative metrics, such as error rate or diagnostic time, should be complemented by clinician and patient feedback [211], because trust, cognitive workload, comfort, and ethical judgment strongly influence safety and acceptance [49, 53, 167, 212]. Domain‐specific criteria and expert review remain crucial when clinical relevance cannot be captured by numerical scores alone [213].

Rigorous evaluation requires comparison with appropriate baselines, including manual procedures, biomechanical aids, and non‐collaborative robotic systems [41, 46, 169]. Such comparisons reveal the added value of collaboration in efficiency, accuracy, and fatigue reduction. For example, HRC‐enabled ultrasound systems can be compared with manual scanning for image quality and examination time [39]. In assistive surgery, shared‐control robots can be benchmarked against passive stabilizers for tremor suppression [46, 169]. These comparisons also help identify trade‐offs, including learning curves and interaction‐induced cognitive load.

In summary, evaluation of the physical brain should balance task performance with clinical outcome quality. Each system should report its intended autonomy level, task boundary, human role, permitted robot actions, escalation triggers, and residual risk. These measures help determine whether robotic execution is safe, reliable, and clinically useful.

Future clinical HRC studies should clearly report the intended autonomy level, human intervention frequency, escalation trigger, task boundaries, and safety endpoints. These details are especially important because the same system may carry different risks in imaging, rehabilitation, surgery, and outpatient care. Evaluation should link quantitative measures, such as latency, diagnostic agreement, intervention rate, safety‐stop rate, and task success, to the specific clinical context rather than implying universal acceptance thresholds [64, 68, 72, 87]. Reports should also distinguish routine confirmation from urgent rescue, as these reflect different oversight models and residual risks. Evaluation should also address equity and deployment robustness. A system that performs well in one hospital, imaging protocol, language group, or patient population may not generalize to settings with different staffing, devices, disease prevalence, or communication needs. Prospective studies should therefore report subgroup performance, missing‐data behavior, handover frequency, and the consequences of delayed or failed escalation [79, 82]. These measures connect technical benchmarks with real clinical safety and help determine whether an HRC system can be responsibly scaled beyond its original development setting [51, 211].

## 6 Future Challenges and Outlook

Despite rapid progress, several barriers remain to be addressed before autonomous and collaborative systems can be deployed safely and widely in real‐world healthcare. These challenges extend beyond technical performance to include trust, accountability, usability, interoperability, cost, and post‐deployment governance.

### 6.1 Challenges

*Robustness in dynamic environments*: Clinical settings are inherently unpredictable, with variable anatomy, patient motion, and environmental disruptions [8, 84]. Robots should operate safely under uncertainty, requiring perception and state‐estimation algorithms that tolerate noise, occlusions, and context shifts.

*Trust and explainability*: Trust is a prerequisite for clinical adoption [25, 47, 50, 211, 214]. Clinicians need confidence in robotic behavior, especially during high‐risk or sensitive interactions [51]. However, many AI‐driven components, such as deep learning models, remain difficult to interpret, raising concerns about unexpected failures [211]. Transparent feedback, calibrated confidence estimates, and timely explanations can help reduce these concerns and improve clinical trust [214].

*Accountability and liability*: As robots assume greater autonomy, responsibility for errors becomes more complex [215]. In shared‐control systems, accountability may be distributed between human oversight and system malfunction, creating legal and ethical uncertainty [212]. Accountability also includes who verifies task boundaries, approves software updates, records clinician overrides, and explains residual risk to patients. Establishing clear governance frameworks for liability, data ownership, and ethical accountability is therefore a pressing challenge.

*Generalizability and interoperability*: Many current systems are designed for specific tasks, devices, or settings, limiting broader use. Scalable deployment requires modular architectures, standardized communication, and adaptive algorithms that integrate with electronic health records and clinical infrastructure [27, 165]. This is also a data‐governance challenge. Autonomous‐clinic systems depend on continuous streams of imaging, sensor, workflow, and patient‐reported data, yet these data are often fragmented across devices, institutions, vendors, and privacy frameworks. Deployment should therefore include privacy‐preserving data infrastructure and clear documentation of training data, software updates, and local monitoring [211].

*Human factors and usability*: Even technically strong systems may fail if they disrupt clinical workflows or increase cognitive workload [28, 211]. Long‐term adoption depends on intuitive interfaces, manageable training demands, and patient comfort, especially in mental health or end‐of‐life care [7, 48, 216]. Usability evaluation should therefore begin early and involve representative clinicians, patients, and care teams, focusing on whether the system fits existing routines, communicates its status clearly, and supports rather than competes with clinical attention. Addressing these issues requires close collaboration among engineers, clinicians, psychologists, ethicists, and implementation scientists.

*Post‐deployment monitoring and adaptive learning*: Clinical HRC systems may change after deployment through software updates, local workflow adaptation, model drift, and evolving user behavior [217, 218]. A system that is safe during validation may perform differently as patient populations, imaging protocols, staffing patterns, or device configurations change. Continuous monitoring is therefore needed to track performance, intervention frequency, escalation events, user overrides, adverse events, and subgroup differences. This is a patient‐safety requirement, not only a technical safeguard [219]. In real clinical settings, unanticipated failures, such as misinterpreting patient movement under unusual lighting or silent performance degradation after demographic shift, can directly lead to missed diagnoses, procedural errors, or physical harm. Post‐deployment monitoring therefore bridges regulatory approval and sustained safe operation by detecting emerging risks before they cause adverse events [220]. If systems learn from new data, updates should be transparent, version‐controlled, and subject to clinical review. Without these safeguards, adaptive autonomy may improve efficiency while gradually weakening safety, auditability, and clinician trust.

### 6.2 Future Directions

The future of HRC in healthcare lies in developing systems capable of dynamically sharing control and decision‐making with human users in a context‐aware manner [6, 7, 28]. Achieving such collaborative autonomy requires real‐time intent prediction, adaptive task allocation, and multimodal feedback integration.

Large‐scale multimodal foundation models may strengthen the professional brain by improving perception, reasoning, and dialogue. Trained on diverse data sources, including medical images, records, physiological, and interaction signals, these models may support more generalizable intelligence across tasks. They may also improve natural language understanding, provide transparent explanations, and adapt to new users or environments with limited retraining [40].

Effective human–AI–robot teams require robots to coordinate technically while respecting human roles, values, and preferences. They should respond to verbal cues, communicate task updates, request assistance when uncertainty increases, and defer to human judgment in ethically sensitive situations [25]. Progress in shared mental models, turn‐taking, and conflict resolution will be critical for fluid and trustworthy teamwork.

Clinical translation also depends on standardized evaluation and clinical validation. Such validation should also include pragmatic endpoints that reflect real deployment, such as failed escalation, staff intervention burden, maintenance requirements, and patient refusal or discomfort. Reporting these endpoints alongside technical accuracy would make clinical HRC studies more comparable and more informative for regulators, hospitals, and future developers. Rigorous clinical trials and collaboration with regulators are required to assess the safety, efficacy, and usability of AI‐powered robots in real‐world settings. Ontology‐driven systems such as HERON may help align robotic behavior with institutional policies and clinical guidelines [45].

Economic feasibility and scalability will be equally important for sustainable adoption. The value of clinical HRC will depend not only on technical performance, but also on whether systems can be maintained, updated, integrated with existing infrastructure, and operated with reasonable training demands. High acquisition costs, maintenance needs, software fees, and workflow redesign may limit adoption, especially in primary care, rural hospitals, and resource‐constrained settings. Future systems should therefore support modular deployment, shared infrastructure and gradual scaling from supervised subtasks to broader workflow support. Reducing cost and training burden will be critical to ensuring that autonomous‐clinic technologies expand access rather than widen existing disparities [216].

Taken together, the main barrier for clinical HRC is not technical performance alone, but the alignment of autonomy with safety, trust, accountability, usability, cost, and governance. Robust perception, adaptive control, foundation models, and natural interaction can expand what robots can do. Broad deployment, however, will depend on whether clinicians can understand system behavior, patients can remain comfortable and protected, institutions can assign responsibility, and health systems can sustain the costs and training demands [152]. Future work should therefore move beyond isolated demonstrations toward risk‐stratified autonomy, standardized reporting, prospective clinical validation, post‐deployment monitoring, and human‐centered workflow design. This pathway could allow autonomous clinics to improve access, consistency, and personalization while keeping human responsibility and the clinician–patient relationship at the center of care.

## 7 Conclusion and Prospects

HRC offers a practical route to clinical autonomy by treating autonomous clinics as supervised care systems rather than machine‐only environments. Across communication, imaging, rehabilitation, surgery, and workflow coordination, robotics can improve precision, consistency, access, and workload while preserving human judgment in diagnosis, consent, escalation, and accountability.

The dual‐brain framework clarifies this route. The professional brain supports clinical reasoning, multimodal interpretation, treatment planning, and patient communication, whereas the physical brain supports embodied sensing, planning, safe control, and task execution. Their integration should remain bounded: autonomy should be matched to task scope, clinical consequence, uncertainty, and the available mechanisms for human oversight and handover.

Future progress will require robustness in unstructured environments, calibrated trust, transparent accountability, clinical validation, regulatory alignment, and human‐centered design. Preclinical studies, simulation, first‐in‐human testing, and deployment monitoring should be connected as a staged evidence chain rather than treated as isolated technical demonstrations.

Personalization will also shape the responsible adoption of clinical HRC. AI‐assisted imaging, surgical planning, robotic execution, and longitudinal monitoring may support individualized screening, treatment planning, delivery, and follow‐up, particularly in diseases with heterogeneous anatomical, functional, or biological trajectories. However, personalization should strengthen multidisciplinary clinical judgment rather than bypass it. It should also account for patient preferences, disability accommodation, language access, demographic performance, auditability, and local workflow constraints.

**FIGURE 4:**
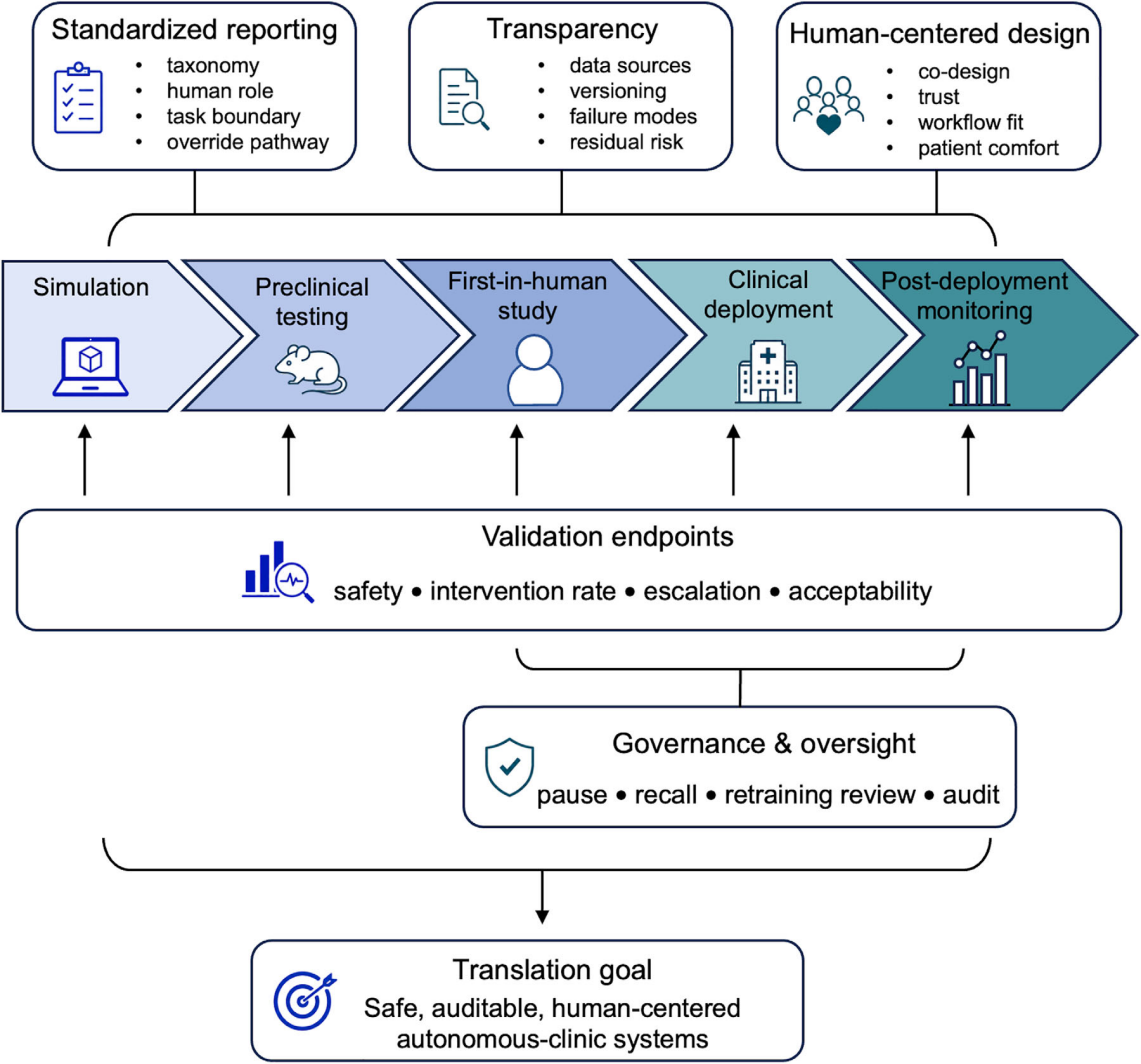
Translational roadmap for clinical HRC in autonomous clinics. The roadmap summarizes key actionable priorities for moving clinical HRC from simulation and preclinical testing to first‐in‐human studies, clinical deployment, and post‐deployment monitoring. Standardized reporting, transparency, human‐centered design, validation endpoints, and governance mechanisms are needed to support safe, auditable, and human‐centered autonomous‐clinic systems.

To translate clinical HRC from promising prototypes to safe autonomous‐clinic systems, future research should move toward standardized, auditable, and human‐centered evidence generation. Figure 4 summarizes this translational roadmap, linking autonomy reporting, staged validation, governance, transparency, and human‐centered design. Key recommendations include (1) standardizing autonomy reporting by applying the four‐level taxonomy and specifying the human role, robot capability, override pathway, and autonomy boundary; (2) expanding validation endpoints beyond task success to include intervention rate, safety‐stop rate, override frequency, failure recovery, workflow burden, and acceptability; (3) connecting simulation, preclinical testing, first‐in‐human studies, and deployment monitoring as a continuous evidence chain; (4) defining post‐deployment protocols for system pause, recall, retraining, software update review, and external audit; (5) improving transparency and accountability by reporting training data sources, version history, known failure modes, responsibility for overrides, and residual risk; and (6) embedding human‐centered design by involving clinicians, patients, ethicists, and workflow managers from early development.

In this restrained sense, collaborative autonomy can support autonomous clinics while keeping human responsibility at the center of medicine. With sustained interdisciplinary effort, HRC could become a foundation for next‐generation clinical automation, improving access, consistency, and personalization without weakening the clinician–patient relationship.

## Author Contributions

X.W. and J.Z. contributed to the study design, data collection and analysis, and the initial and final drafts of the manuscript. M.X., H.C., P.Z., X.C., B.L., and D.S. contributed to the management and coordination of the project and helped review the manuscript. D.S. and M.H. contributed to study design, supervision, manuscript review, and editing. All authors performed a critical review and approved the final manuscript and important intellectual input.

## Funding

D.S. and M.H. disclose support for the review and publication of this work from the RCSV seed fund Eye Robot for Autonomous Clinic: Prototype Development (P0057912) from PolyU.

## Conflicts of Interest

The authors declare no conflicts of interest.

## Ethics Statement

The authors have nothing to report.

## Data Availability

The authors have nothing to report.
